# 

**Published:** 1894-02-24

**Authors:** 


					T8fl HOSPITAL. ' "vj 0 February 24th, 1894.
? PRICE TWOPENCE. IBSS-ii:, WW "fesa WITH EXTRA NURSING SUPPLEMENT.
/No. 387, Vol. XV.?Feb. 24th, 1894. llni 8a- ea"
^Wiatered at the General Post Office as a Newspaper for <-?JMCj -
transmission in the United Kingdom and Abroad.! - ~~s^/
Monthly Farts, 6d.; post free, Bd.
transmission in the United Kingdom and Abroad.] Ditto per Annum, 8s., post free.
No. 387. Vol XV. WITH EXTRA NURSING SUPPLEMENT. Saturday,
February 24th, 1894.

				

